# Intracellular complement: Evidence, definitions, controversies, and solutions

**DOI:** 10.1111/imr.13135

**Published:** 2022-09-13

**Authors:** Ben C. King, Anna M. Blom

**Affiliations:** ^1^ Department of Translational Medicine Lund University Malmö Sweden

**Keywords:** beta‐cells, C3, CD59, intracellular complement, microbial infections

## Abstract

The term “intracellular complement” has been introduced recently as an umbrella term to distinguish functions of complement proteins that take place intracellularly, rather than in the extracellular environment. However, this rather undefined term leaves some confusion as to the classification of what intracellular complement really is, and as to which intracellular compartment(s) it should refer to. In this review, we will describe the evidence for both canonical and non‐canonical functions of intracellular complement proteins, as well as the current controversies and unanswered questions as to the nature of the intracellular complement. We also suggest new terms to facilitate the accurate description and discussion of specific forms of intracellular complement and call for future experiments that will be required to provide more definitive evidence and a better understanding of the mechanisms of intracellular complement activity.

## 1 INTRODUCTION

The traditionally understood roles of complement in innate immunity take place in the extracellular environment, directly opsonizing and even lysing some pathogens via the action of C3 deposition and formation of the terminal membrane attack complex^1^ but also acting as a danger sensing mechanism, transmitting signals to cells via cell‐surface receptors of activated complement components.^2^ While these functions have largely been attributed to circulating complement proteins secreted mainly from the liver, it has been recognized that local expression of complement proteins can also have an important role in tissues,^3^ for example, in infection^4^, ^5^ and in complement‐dependent pathologies.^6^, ^7^, ^8^ The discovery that some cell types express multiple complement proteins required for complete activation pathways, leading to spontaneous activation, also opened the possibility for autocrine complement activation playing roles in cellular homeostasis, activation, and inflammation.^9^, ^10^ For example, more recently, cell‐intrinsic C3 expression in fibroblasts has been shown to play an important role in priming tissue sites for inflammation.^11^ In autoinflammatory disease, repeated attacks can increase in severity. It was shown that mice injected at the same site with identical doses of sterile inflammatory danger‐ or pathogen‐associated molecular patterns (DAMPs or PAMPs) suffered increasing levels of inflammation, while injection at different sites did not. This was not dependent on the adaptive immune system, as shown using SCID and RAG1‐KO mice, but was dependent on C3, as shown using C3‐KO mice. In addition, adoptive transfer of exposed fibroblasts from WT but not C3‐KO mice “primed” the recipient site for increased inflammation, indicating the importance of fibroblast‐expressed C3 in this priming process. This detailed and clear study indicates a role for local, or cell‐intrinsic C3 in fibroblasts in sterile inflammation. The exact function of C3 was not however defined, although global C3aR‐KO mice did not undergo the same priming process, indicating a role for C3 cleavage and C3a production. The authors did find that increased C3 expression in primed fibroblasts was associated with increased aerobic glycolysis and cell‐surface expression of the glucose transporter GLUT1, both of which have been linked to C3‐mediated signaling in T‐cells^12^, ^13^ and a pro‐inflammatory phenotype. Consistent with this, primed fibroblasts with increased aerobic glycolysis secreted larger amounts of inflammatory cytokines, and inhibition of glycolysis in vivo prevented the fibroblast/C3‐dependent priming of inflammation,^11^ demonstrating the link between cell‐intrinsic C3, and immunometabolism. While this is an important paper that clearly demonstrates a clear role for cell‐specific C3 expression by fibroblasts in inflammation, it does not address the details of where and how C3 activation is taking place, whether within the cell or the extracellular space and what C3 is being cleaved by. Other recent work has, however, pointed to the existence and function of individual complement proteins as well as complement pathways within the intracellular boundaries of the limiting cell membrane. This review will summarize the evidence for intracellular functions of complement, highlight controversies over published data, attempt to define what we should refer to as “intracellular complement,” and suggest experimental solutions to clarifying these issues.

## 2 INTRACELLULAR COMPLEMENT: SPECIFIC COMPONENTS OR COMPLETE PATHWAYS?

One of the questions raised following the discovery of intracellular complement is whether specific complement proteins play individual intracellular roles that may differ from canonical extracellular functions, or whether complete and functional canonical complement pathways exist within the cell. In the first case, individual complement proteins may play novel intracellular roles, for example, by interacting with intracellular ligands not found in the extracellular environment. In the second case, entire canonical complement convertases or pathways may exist and be activated within the cell, most likely in membrane‐limited compartments such as endosomes. These proteins could conceivably originate from the cell itself, or be taken up from the extracellular environment. Some of the evidence for both canonical and non‐canonical functions of individual intracellular complement proteins are summarized in Table 1 and described below.

**TABLE 1:** Non‐canonical sites, sources, and roles of selected intracellular complement proteins.

Protein	Source: Exogenous or cell‐intrinsic	Role	Site of action	References
CD59 (IRIS isoforms)	Cell‐intrinsic	Responsive exocytosis of insulin in beta‐cells	Cytosol/insulin granules	[16]
C1Q	Exogenous	Regulation of CD8+ T‐cell mitochondrial metabolism	Mitochondria	[42]
Factor H	Cell‐intrinsic	Tumor progression	Lysosomes	[43]
C5aR	Cell‐intrinsic (receptor)	Regulation of monocyte/macrophage immunometabolism	Mitochondrial surface	[50]
C3aR	Cell‐intrinsic (receptor)	Inhibition of mitochondrial metabolism in oxidative stress	Trafficks from cell surface to mitochondria	[52]
C3/C3a	Cell‐extrinsic	Pathogen detection	Cytosol	[22]
	Cell‐extrinsic	Xenophagy	Cytosol	[23]
	Cell‐intrinsic	Autophagy regulation	Cytosol	[25]
	Cell‐intrinsic	Pathogen detection	Cytosol	[28]
	Cell‐intrinsic/extrinsic	Lung epithelial cell survival under oxidative stress	Stored in endosomes	[37]
	Cell‐intrinsic	T‐cell homeostasis	Endosomal	[31]
	Cell‐intrinsic	T‐cell activation		[59]

### 2.1 Evidence of non‐canonical intracellular functions of individual complement proteins, independent of complement activation cascades

#### 2.1.1 Intracellular roles of CD59

We have previously found that CD59, the GPI‐anchored cell surface MAC inhibitor, is required for insulin secretion from beta‐cells.^14^ This surprising finding pointed to a clear non‐canonical function for this complement inhibitor. While siRNA‐mediated knockdown of CD59 led to a failure of clonal beta‐cells to secrete insulin in response to glucose, removal of cell‐surface CD59 by enzymatic cleavage of the GPI anchor did not affect insulin secretion, suggesting that an intracellular pool of CD59 was involved. Within the cell, CD59 co‐localized and co‐immunoprecipitated with soluble N‐ethylmaleimide‐sensitive factor attachment protein receptors (SNARE proteins), which form part of the exocytosis machinery, providing a mechanism by which CD59 is involved in insulin secretion. Targeted mutagenesis of CD59 cDNA constructs revealed that removal of the C‐terminal GPI anchor attachment site did not affect insulin secretion, but allowed retrotranslocation of CD59 from the ER to the cytosol, where interaction with exocytotic machinery could take place,^15^ again showing that non‐canonical forms of CD59 lacking a membrane anchor, and therefore unable to inhibit complement, were able to mediate insulin secretion. We have since verified the existence of endogenous splice variants of human CD59 that are expressed in pancreatic islets. Although the overall structure of the CD59 globular domain is conserved in these splice isoforms, these variants have unique C‐terminal domains, and lack a C‐terminal GPI anchor, resulting in isoforms that traffic to the cytosol via retrotranslocation from the ER, after glycosylation. These cytosolic isoforms associate with insulin granules, and mediate insulin secretion.^16^ Whereas canonical CD59 is ubiquitously expressed at the cell surface, the novel isoforms (named CD59‐IRIS, isoforms rescuing insulin secretion) have a tissue‐specific pattern of expression. This is reflected in the mouse, where CD59 has been duplicated, resulting in the ubiquitously expressed CD59A, as well as CD59B, which has expression restricted to certain tissues. It has been described that mouse CD59B expression is predominantly found in the testes,^17^ but many other sites were not specifically examined. We found that in mouse MIN6 clonal beta‐cells, CD59B had dominant expression over CD59A,^16^ which could be missed if looking at the total pancreas, of which pancreatic islets make up only about 1% of the total mass. The mouse CD59‐IRIS isoforms are also found only as splice products of CD59B, and not CD59A, suggesting that the tissue‐limited expression of mouse CD59B is due to the specialized function of the IRIS isoforms.

The fact that CD59 has differing structural requirements for its divergent extracellular and intracellular functions is highlighted by human mutations. An amino acid substitution at position 64 of the mature protein replaces cysteine with a tyrosine, preventing the formation of a disulfide bond and causing misfolding of the protein. While this mutant form of CD59 does reach the surface, it is not recognized by conformation‐specific anti‐CD59 antibodies, and cannot regulate complement,^18^ leading to a devastating complement‐dependent neurodegenerative pathology.^19^ However, none of these patients were described as insulin‐deficient, and we have shown that the C64Y mutation does not affect the ability for CD59 to allow insulin secretion,^15^ The substitution of the cysteine residue at position 64 for a tyrosine prevents the formation of a disulfide bond, altering the structure of cell‐surface CD59 and preventing interaction with and inhibition of MAC. However, this substitution does not affect insulin secretion, possibly reflecting the fact that this function occurs in the cytosolic environment, which has an overall reducing redox potential, and where disulfide bonds are therefore less important for controlling protein structure. This probably also reflects different active sites of human CD59 for MAC inhibition and for insulin secretion: while the central portion of the CD59 sequence is required for MAC inhibition,^20^ we hypothesize that the insulin secretion function is contained within the N‐terminal region, based on comparison of CD59‐IRIS from different species. It would still be of interest to determine folding and verify cellular localization of the C64Y mutant, as well as to verify interaction with SNARE proteins as has been found for the non‐mutated CD59 isoforms. We did however find that the N‐glycosylation site of CD59 is required for the retrotranslocation of CD59‐IRIS to the cytosol,^15^ and therefore for its function in insulin secretion, whereas the same glycosylation site on the canonical cell‐surface CD59 does not appear to play any role in MAC inhibition.^20^

CD59 therefore gives a clear‐cut example of a complement protein with dual roles in different cellular compartments, firstly in complement inhibition at the cell surface, and secondly within the cytosol in insulin secretion via interaction with SNARE proteins. This sets a precedent as a multifunctional complement protein with alternative sub‐cellular locations and functions, which is regulated by tissue‐specific alternative splicing patterns.

#### 2.1.2 Intracellular roles of C3

The main focus of intracellular complement research has been on C3, the central component of complement, for which there is evidence of intracellular involvement in pathogen sensing, as well as homeostatic roles in cell survival and metabolic control. Several papers have demonstrated functionally relevant interactions of C3 or C3 activation products with cytosolic proteins, setting a precedent for the description of intracellular C3 interactions. The first major paper showed that when C3 is carried into a cell after deposition on the surface of a cytoinvasive pathogen, it can trigger cell‐autonomous immunity, in the form of NFkB and IRF signaling.^21^ The authors showed that C3‐opsonized non‐enveloped virus particles triggered these pathways in non‐immune cell lines from multiple species, in a manner partially dependent on mitochondrial antiviral signaling protein (MAVS), resulting in cell‐intrinsic immunity and pro‐inflammatory cytokine production. Furthermore, Sorbara et al also showed that cytoinvasive bacteria that are opsonized by C3 prior to infection are targeted by xenophagy, a form of pathogen‐targeted autophagy, due to an interaction between opsonized C3 and the cytosolic protein ATG16L1,^22^ a key protein involved in the recruitment and targeting of the autophagy machinery. These papers demonstrate clear intracellular roles for C3 when activated C3(b) is brought into the cytosol by invasive pathogens. Although the exact forms of C3 responsible for the interactions described in these papers were not defined, it can be assumed that activated C3b/iC3b/C3d would be responsible, and attached to the invasive pathogen surface. These roles are parallel to the well‐established extracellular roles of C3 in danger/pathogen detection, where the conformational change triggered by activation of C3 to C3b reveals cryptic sites that interact with new ligands.^23^ While the exact cytosolic binding partners and mechanisms of interaction of C3 in these newly identified intracellular roles are still not clear, the concept of extrinsically activated C3 acting as a danger signal in the intracellular environment is a logical extension of its known extracellular role.

There is however more of a philosophical leap in the argument that cell‐intrinsic C3, expressed and stored within the cell, plays homeostatic intracellular roles in the absence of infection or danger‐sensing. While this may represent a change in the apparent role of C3, it could still be making use of the ability for C3 to act as an inducible switch, whereby cleavage in response to a certain stimulus (possibly context‐ or tissue‐specific) causes a conformational change, allowing interaction with novel ligands and triggering of the desired response. In this scenario, C3 may not be acting as a danger signal that can be triggered by the recognition of DAMPS or PAMPS by pathogen recognition receptors of the complement system. Rather, C3 may be acting in non‐canonical roles via interaction with a separate set of non‐complement ligands specific to the intracellular environment. For example, the C3/ATG16L1 interaction, identified in the triggering of xenophagy of cytosolic bacteria, has also been demonstrated to have a homeostatic role in the regulation of autophagy. C3 is highly expressed in human pancreatic islets, and C3‐knockout beta‐cell lines demonstrated a distinct defect in autophagic turnover in a gene‐dose‐dependent fashion,^24^ with an accumulation of non‐acidified immature autophagosomes. An accumulation of lipidated LC3‐II, an autophagosomal marker, was also found in the pancreatic islets of C3‐knockout mice. These defects could not be rescued by the addition of extracellular purified C3 or C3a, indicating the requirement for an intracellular source of C3, and were not replicated by knock‐down of C3aR1, suggesting a non‐canonical role of C3, separate from conventional C3 activation and signaling. C3‐knockout autophagy‐defective beta‐cells also underwent increased apoptosis when faced with diabetogenic cell stresses such as gluco‐ and lipo‐toxicity, consistent with a known protective role of autophagy in beta‐cells,^25^ and demonstrating a role of C3 in cytoprotective homeostasis. C3 expression was also upregulated in pancreatic islets from human donors with type 2 diabetes (T2D) compared to healthy donors,^24^ and has been recently identified as one of a set of just 10 differentially expressed genes in pancreatic islets that accurately predict T2D status.^26^ The independently confirmed interaction between C3 and ATG16L1, and a defective autophagic phenotype in C3‐knockout cells, suggests that endogenous C3 must be found within the cytosol in this cell type, to interact with ATG16L1. This raises questions as to how C3, a typically secreted protein, should enter the cytosol, what conformation the protein will have in the cytosolic environment, and what other complement factors, if any, it interacts with here.

Some answers may have come in a recent paper, which assesses the routes that C3 may take to enter the cytosol.^27^ Using cellular fractionation techniques, processed C3 was found in the reducing environment of the cytosol, as also confirmed using confocal microscopy of C3 fused with a redox‐sensitive GFP mutant. This suggests that C3 can be retrotranslocated from the ER directly into the cytosol, a finding also backed up by expressing C3 variants tagged with a biotin‐acceptor peptide, which becomes biotinylated only when reaching the cytosol in cells co‐expressing the cytosolic BirA enzyme.^28^ As well as retrotranslocation, the use of alternative translational initiation sites has been suggested as providing a means for C3 to be expressed directly into the cytosol.^24^, ^27^ This can be modeled by site‐directed mutagenesis of cDNA constructs, or CRISPR/Cas9‐mediated genome editing, to remove the initial ATG translational start site, to produce “∆ATG1” cells lacking a functional canonical C3 translational start site. In these cases, the C3 protein was still expressed from a start site downstream of the signal peptide. Lacking the signal peptide that normally directs the nascent protein into the ER and secretory pathway, this resulted in a pro‐C3‐like 180 kDa unglycosylated protein found within the cytosol, but absent from the ER, secretory pathway, or the extracellular space.^27^ Functional evidence for this protein has been found in intracellular detection of invasive *Staphylococcus aureus* in gene‐edited alveolar epithelial A549 cells; these bacteria became opsonized with a novel 65 kDa product of the C‐terminal region of the C3 alpha‐chain, as mapped by epitope‐specific monoclonal antibodies. This deposition occurred in WT cells but also in gene‐edited ∆ATG1 cells only expressing cytosolic C3,^27^ therefore also attributing deposition of this fragment in WT cells to a cytosolic isoform. Further gene editing was carried out to completely excise exons 2–25 of the C3 gene, leaving an in‐frame product that still expressed a similar‐sized C3 alpha‐chain fragment, which also became associated with infecting *S. aureus* cells, verifying this function. This 65‐kDa C3 product was not produced when *S. aureus* were incubated with human serum, nor when purified C3 was incubated with *S. aureus* proteases, but it was also produced when *S. aureus* was incubated with C3‐containing cytosolic fractions of human hepatocytes. Together, these results demonstrate that cytosolic intracellular C3 is cleaved to a novel 65 kDa product by cytosolic factors in the presence of cytoinvasive *S. aureus*. This fragment does not contain the thioester group that mediates covalent attachment of C3 to surfaces but does contain residues essential for binding to CR2. Consequently, although bacterial survival within gene‐edited A549 cells was not affected, their killing by phagocytes once recovered from the epithelial cells was strongly linked to the deposition of the 65‐kDa product, with those recovered from C3‐KO cells experiencing increased survival.^27^ While this again indicates a familiar role of C3 in pathogen detection, the difference from canonical complement is in the source of C3, in this case being cell‐intrinsic and cytosolic. In addition, the cytosolic factors responsible for facilitating its deposition onto intracellular bacteria are not yet identified. These results show that cell‐intrinsic cytosolic C3 may have further important roles in intracellular pathogen detection and cell‐intrinsic cellular immunity, involving non‐canonical mechanisms of C3 activation.

Additional potential roles of C3 in homeostatic roles have been identified in immune cells, as reviewed elsewhere.^29^ It has been shown that several cell types appear to contain intracellular stores of C3 or C3a,^30^ within intracellular vesicles co‐staining for endosomal markers and that human C3 can be cleaved into bioactive C3a and C3b by lysosomal cathepsin L, verified by co‐incubation of purified proteins.^30^ The resultant production of C3a supported T‐cell activation and inflammatory cytokine production, which was increased in T‐cells from patients with autoimmune arthritis, whereas cathepsin L inhibition treatment of primary cells reversed their hyper‐inflammatory phenotype. The hypothesis presented is that C3‐dependent C3a production is required for normal T‐cell homeostasis as well as activation and that this intracellular function of C3 may be disconnected from the function of secreted C3. In support of this, three human patients with primary serum C3 deficiencies were shown to have normal levels of intracellular C3a, as detected by intracellular flow cytometry and confocal microscopy using a C3a neo‐epitope specific antibody.^30^ This raised criticism as to how a primary deficiency serious enough as to completely prevent protein secretion, could still allow expression and function of intracellular C3. One answer could lie in the example of CD59, described earlier; CD59 mutations preventing cell‐surface localization, or preventing MAC inhibition, do not necessarily interfere with the unrelated intracellular function of mediating insulin secretion.^15^ In the case of the C3 mutations in question, the first was a single amino acid Ser550Pro substitution within the beta‐chain,^31^ presumably causing protein misfolding that prevents secretion. The open reading frame is otherwise unaffected, and therefore an intact pro‐C3 protein is predicted to be expressed, that, although it is not secreted, could hypothetically still be a substrate for intracellular cleavage and therefore a source for intracellular C3a generation. The evidence suggested cathepsin L‐mediated cleavage of C3 within lysosomes, which are typically at low pH (4.5–5), in which case the normal folding of C3 may be in any case disrupted. The second and third patients were siblings with deletions within the beta‐chain, although this was not examined in detail; if the deletion did not cause a frameshift (a chance of 33%), the resultant expressed protein could also be a potential source for the proposed lysosomal generation of C3a, even if protein structure is disrupted enough to prevent secretion. The potential for the products of these human C3 mutants to produce C3a by cathepsin cleavage has so far not been directly addressed experimentally, and so the question remains unresolved.

Other intracellular roles of cathepsin L‐mediated C3 cleavage have also been suggested. It has been shown that factor H actively taken up by apoptotic cells acts as a cofactor for cathepsin L‐mediated C3 cleavage, increasing iC3b opsonization of apoptotic cell surfaces, and diminishing the inflammatory potential during apoptotic cell clearance.^32^ Serum‐purified factor H greatly enhanced the cleavage of C3 by purified cathepsin L in vitro, and all three proteins co‐localized within apoptotic cells. In this scenario, cathepsin L cleaves and activates cell‐intrinsic C3 and allows its deposition on the apoptotic cell surface, presumably triggered during the process of apoptosis induction, either by fusion of C3‐containing endosomes and cathepsin L‐containing lysosomes, or by the leakage of their contents into the cytosol during apoptosis induction,^33^ allowing their interaction. In addition, cathepsins have been demonstrated to cleave C3 intracellularly within hypoxic gut epithelial cells, causing anaphylatoxin release and contributing to inflammation during ischemia,^34^ and cathepsin D was also recently identified as directly cleaving C5 within lysosomal/endosomal compartments in colonic epithelial cells, driving tumorigenesis via C5aR1 signaling.^35^

Cell‐intrinsic (i.e., de novo synthesized, not internalized) C3 stores have also been identified by others within endosomes of lung epithelial cells,^36^ which increased after exposure to inflammatory cytokines, and in primary cells from patients with various lung diseases. The presence of intracellular C3 stores contributed to cellular survival under hypoxic conditions, as demonstrated by the use of CRISPR/Cas9 C3 knockout, as well as siRNA‐mediated C3 knockdown. Cell‐intrinsic production and storage of C3 may therefore be a protective mechanism that allows autocrine, or intracellular “intracrine” signaling, contributing to cellular activation or survival, when C3 is cleaved in response to external factors such as hypoxic conditions or cellular stress.

Other roles of intracellular C3 have been shown in B lymphocytes. These cells do express endogenous C3, but the majority of intracellular C3 was found to be taken up from the extracellular environment.^37^ C3a was also found intracellularly, but only when cells were incubated with serum, and not with purified C3, factor B‐depleted serum, or serum in the presence of EDTA, showing that this intracellular C3a was produced via the action of alternative pathway convertases sourced from the extracellular environment, and not by intracellular proteases, at least under the conditions studied. After uptake into B cells, C3 trafficked to the nucleus, where it interacted with chromatin, mediated by a direct non‐ionic interaction between C3a and DNA.

#### 2.1.3 Intracellular roles of C1q

C1q is the soluble pattern recognition receptor of the classical pathway of complement, but is also a member of the C1q/tumour necrosis factor superfamily,^38^ suggesting an evolutionary history in cytokine‐like functions. Members of this family share a similar homotrimeric globular domain structure, which for C1q makes up the globular heads of C1q containing the IgG‐binding regions. A cellular receptor for this globular domain, p32/gC1qR, is found on the surface of many cell types, and C1q binding can induce cell‐specific biological responses.^39^ gC1qR is however predominantly found within mitochondria, and the homozygous knockout is embryonic lethal, due to failure of mitochondrial protein synthesis.^40^ Recently, it has been shown that C1q attenuates the function of memory precursor effector CD8^+^ T‐cells via regulation of mitochondrial metabolism.^41^ On surface binding of C1q globular domains, both the gC1qR receptor and C1q are internalized and traffic to mitochondria. *C1qa* knockout mice had stronger antiviral T‐cell responses, as well as stronger lupus autoantibody responses in a mouse model of SLE, which was reversed by depletion of CD8^+^ T‐cells.^41^ CD8+ memory precursor effector cells from C1qa knockout mice also had decreased total mitochondrial mass and spare respiratory capacity. This was not the case in C3 knockouts, showing that C1q affects CD8^+^ T‐cell function via a direct effect on their mitochondrial function, in a manner independent of C3, and therefore independently of canonical complement pathways. Importantly, CD8^+^ T‐cells from *C1qa*‐KO mice responded normally when transferred into WT mice, and WT CD8^+^ T‐cells had exaggerated responses in *C1qa*‐KO mice, indicating that a cell‐extrinsic source of C1q is responsible for these functions. These data therefore show that exogenous C1q can be taken up by CD8+ T‐cells, where it mediates intracellular metabolic regulation via interaction with mitochondrial gC1qR. It is yet to be investigated whether other functions mediated by C1q binding are mediated by similar uptake and mitochondrial interaction in other cell types.

#### 2.1.4 Intracellular roles of Factor H, and related proteins

Factor H expression was found to be negatively associated with disease outcomes in renal cell carcinoma and lung adenocarcinoma.^42^ Interestingly, this association was strongest with intracellular staining of factor H, rather than secreted factor H associated with the cell membrane, where factor H interacts with sialic acid on the surface of host cells.^43^ Although factor H expression regulated extracellular complement activation, factor H knockdown by siRNA, but not the addition of factor H‐blocking antibody, altered cellular phenotypes, despite both treatments having a similar effect on extracellular C3 cleavage. This shows that an intracellular function of factor H was responsible for the reported changes in gene expression, cellular growth and migration, and p53 suppression that was associated with factor H expression. In comparison with the effects of factor H silencing, no difference in tumor growth was seen when cells were inoculated into factor H‐deficient mice, demonstrating the cell‐intrinsic nature of this function of factor H. In addition, the function was cell‐type specific, with no effect of factor H knockdown in other cell types, also ruling out non‐specific off‐target effects of siRNA treatment. Interestingly, intracellular factor H co‐localized with both late endosome/lysosomal markers and C3, where it may act as a co‐factor in C3 cleavage by lysosomal enzymes.^32^ Further work will be required to investigate the mechanisms by which factor H is affecting the outcome of adenocarcinoma and renal cell carcinoma, and whether this is occurring via interaction with C3 and regulation of C3a production.

Complement factor H‐related 3 (FHR‐3) is one of a family of factor H‐related proteins thought to have arisen by duplication of the factor H gene.^44^ When added to retinal pigmented epithelial (RPE) cells, FHR‐3 bound to oxidative stress epitopes and was internalized, triggering upregulation of complement component expression, and intracellular C3a production,^45^ which appeared first diffusely throughout the cytosol, rather than in any distinct organelle, but after 6 hrs translocated to the cell membrane. Associated with this induced C3 activation, the RPE cells subsequently secreted increased levels of pro‐inflammatory cytokines. This has clear implications for age‐related macular degeneration (AMD), a common eye disease with strong links to oxidative stress and complement activation, and for which loss of FHR‐3 deletion is protective,^46^ although again, the underlying mechanism requires further investigation, to determine whether the effects of FHR‐3 are entirely C3‐dependent, to verify whether intracellular or extracellular C3 activation is occurring within the RPE, and by which mechanism C3 may be being cleaved, and C3a generated.

There have been reports of other complement proteins acting intracellularly, such as C1s being recently identified as a prognostic marker in renal cancer, where expression was linked to poorer prognosis, a more aggressive cancer cell phenotype, and possibly increased C4d generation.^47^ However, the prognostic value of C1s expression was not linked to extracellular complement deposition, silencing C1s in vitro reduced cellular proliferation, and extracellular addition of C1s to non‐expressing cancer cells did not alter their phenotype, suggesting a cell‐intrinsic role outside of the complement cascade. Further work is therefore required to fully investigate the intracellular interactions of complement proteins, and to determine whether they are acting in canonical or non‐canonical fashions.

### 2.2 Evidence for intracellular canonical functions of complement

As mentioned above, evidence exists that individual extrahepatic cells^3^ and tissues^48^ can express all the required components for the building of complement convertases, and therefore could produce complement activation products and anaphylatoxins that act in an autocrine or paracrine manner, for example via activation of the alternative pathway in individual cell types.^9^ In recent years though, evidence has also emerged that canonical complement pathways could also be activated within the intracellular environment. Studying intracellular canonical functions of complement already presents several challenges, in being able to determine or differentiate between cell‐intrinsic production of complement factors, compared to uptake of extracellular complement proteins. This provokes the question as to how to differentiate between activation of complement proteins internalized from the external environment, compared to cell‐intrinsic production of convertases or anaphylatoxins, or whether both can occur interchangeably, especially in cases of auto‐ or paracrine complement activation. More work is required in the details of these pathways, and this will be discussed further below.

The most recent major paper providing evidence for intracellular canonical complement pathways argued that intracellular C5a signaling was required for the activation of primary human macrophages and cytokine production in response to sterile inflammation.^49^ Three main strands of evidence were provided for the intracellular nature of this complement activation. First was the detection and co‐localization of both C3b and Bb within macrophages by confocal microscopy, suggesting the formation of intracellular alternative pathway convertases for C3, and therefore potentially C5. This conclusion depends upon the validation of the neo‐epitope‐specific antibodies used, and their recognition of only activated cleaved Bb and C3b, but not the intact uncleaved proteins, although the cleavage of factor B to Bb was also verified by western blot. Second, the presence of intracellular convertases was further evidenced by inhibition of cytokine production in stimulated macrophages after the addition of cell‐membrane permeable factor B inhibitor, but not an inhibitory anti‐factor B antibody that could not enter the cell. Thirdly, the intracellular nature of resultant C5aR signaling was explained by the surprising identification of C5aR on the surface of mitochondria. The activation of these primary macrophages was also inhibited by a cell‐membrane permeable C5aR antagonist, but not by an antagonist that did not enter cells, further supporting the intracellular nature of the signaling. In addition, isolated mitochondria exposed to C5a demonstrated increased activity as shown by increased production of reactive oxygen species, which was also blocked by C5aR antagonists, again tying intracellular complement signaling into the concept of cellular immunometabolism, whereby the inflammatory or activatory potential of immune cells is linked to switches in biochemical pathways of cellular energy metabolism.^50^ Indeed, stimulation of human monocytes resulted in a shift towards aerobic glycolysis, typical for pro‐inflammatory activation, which was also inhibited by the cell‐permeable C5aR antagonist.^49^ Importantly, this paper is the first direct evidence of the formation and function of intact intracellular canonical complement convertases. While the previous focus in the field has been on intracellular cleavage of C3, it is important to remember that an increase in the ratio of C3b subunits switches the specificity of complement C3 convertases to C5 cleavage, and so the presence of an intracellular C3 convertase also opens the possibility for C5a production. Currently, there are no reliable methods for distinguishing between C3 and C5 alternative pathway convertases, as they contain the same constituent proteins.

As well as the identification of C5aR on mitochondria in macrophages, a separate group has also identified C3aR on mitochondria in RPE cells,^51^ whereby cell‐surface C3aR was internalized and trafficked to mitochondria in response to oxidative stress. C3a addition increased calcium uptake in isolated mitochondria and inhibited mitochondrial respiration in a manner that was inhibited by a specific C3aR antagonist. In addition, they found that the mitochondrial J‐haplotype, which is linked to altered nuclear complement gene expression^52^ and conveys an increased risk of AMD,^53^ a disease with a strong link to complement activation,^54^ had increased basal levels of C3aR in the absence of oxidative stress. The source of C3a, whether extracellular or intracellular, was not assessed in this paper, although RPE cells express many complement components themselves.^48^ Nevertheless, the data demonstrates a potentially important role for C3aR on mitochondria in the RPE, in regulating mitochondrial function and subsequent cellular fitness in conditions of complement activation. Conditions that may induce C3aR and C5aR trafficking to, or function on mitochondria, should also be assessed in other cell types.

There is therefore evidence that the formation of canonical convertases, production of anaphylatoxins, and C3aR/C5aR signaling can occur entirely intracellularly, within distinct compartments. Whether this may happen independently of, or downstream of non‐canonical C3 cleavage, for example by cathepsins, remains to be investigated.

## 3 CONTROVERSIES REGARDING INTRACELLULAR COMPLEMENT

### 3.1 Where does it come from?

Intracellular complement proteins can clearly only come from two sources: the extracellular environment, an *extrinsic* source, or through protein expression by the cell itself, an *intrinsic* source. Apart from the complement receptors and cell‐surface inhibitors, complement proteins are largely seen as secreted proteins found in the circulation. Secreted proteins typically have a signal peptide that, as the nascent protein is produced from the ribosome, directs entry of the growing polypeptide through the Sec61 pore within the membrane of the endoplasmic reticulum (ER).^55^ Within the ER lumen, the protein folds, with chaperones such as disulfide isomerase helping the formation of correct disulfide bonds. Within the ER and Golgi apparatus, proteins also undergo post‐translational modifications, such as the furin cleavage of pro‐C3 into separate alpha‐ and beta‐chains,^56^ and the addition of N‐linked glycosylation groups. Secreted proteins then traffic via secretory vesicles to the cell surface and are released to the extracellular environment.

A C3 uptake and recycling pathway has also been identified,^57^ by which cells take up hydrolyzed C3 from the extracellular environment, and it is therefore difficult to identify whether C3 within a cell is from a truly intrinsic, or extrinsic origin. The providence of intracellular C3 can be deduced in several ways. Firstly, pro‐C3, unprocessed into separate alpha‐ and beta‐ chains and therefore distinguishable from mature C3 on reducing SDS‐PAGE, can be only of an intracellular origin, as normally this is not secreted into the extracellular space to be then taken up. Secondly, if the cells in question contain no C3 mRNA, and therefore do not express any of their own C3 protein, then it is logical that any intracellular C3 stores have been taken up from the extracellular environment. This is also experimentally achievable by creating CRISPR/Cas9‐mediated knockout cells, isolating cells from C3‐KO mice, or using siRNA‐mediated knockdown. The release and subsequent re‐uptake of C3 or other complement proteins could also be studied by incubating cells with labeled proteins, or the use of inhibitors of secretion or protein uptake. Several papers have found that resultant phenotypes caused by knock‐down or depletion of complement components at the genetic level cannot be rescued by the presence or addition of these individual components in the extracellular environment, suggesting that an endogenous source is required, and therefore implicating separation of pools of complement proteins from endogenous and exogenous sources, for example, C3 in regulated autophagy within beta‐cells,^24^ factor H in renal cell carcinoma,^42^ or addition of C3a to human T cells where endogenous production was prevented by cathepsin L inhibition.^30^ In this last case, the phenotype of T cells from patients deficient in LFA‐1, which cannot properly upregulate C3 and therefore generate intracellular C3a required for efficient Th1 effector responses, were also not rescued by the addition of C3a into the medium, but did show increased IFN‐gamma production when treated with C3a‐encoding adenovirus or electroporated with C3a‐encoding mRNA,^58^ again demonstrating a difference in the function of endogenous cell‐intrinsic, compared to cell‐extrinsic, complement components. This is not always the case, however, as stores of C3 identified as contributing to survival in lung epithelial cells exposed to hypoxic conditions, could be restored by the addition of C3 to the culture medium.^36^ The role of C3 and other complement proteins may therefore be cell‐type specific.

As described above, some descriptions of intracellular C3/C3a find it within endosomal compartments in cells,^30^, ^36^ which suggests an extracellular source internalized by endocytosis, although many markers of endosomes are imperfect and overlap with other intracellular organelles, or fail to distinguish between different subtypes of endosomes.^59^ However, these cells also synthesize their own de novo C3, and contain pro‐C3 as a result, so it cannot be ruled out that endosomal stores of C3 do not come from a cell‐intrinsic source.

In addition to the proposed roles of C3 and intracellular complement in cell viability, intracellular pathogen detection can occur via pathogen‐associated extrinsic C3^21^, ^22^ or also by cell‐intrinsic endogenous C3 that becomes activated on the invasion of pathogens into the cytosol.^27^ This raises questions as to how cell‐intrinsic C3, a well‐known secreted protein, should enter the cytosol.

### 3.2 C3 presence in the cytosol: how does it get there?

The cytosol refers to the gel‐like fluid filling the intracellular space, within which organelles are suspended. While proteins secreted by the canonical secretory pathway are synthesized into the ER and trafficked via vesicles to the cell surface, they are not normally expected to cross the limiting membranes of these organelles and enter the cytosol. Relevant cytosolic functions of C3 have been demonstrated in the case of the invasion of pathogens into the cytosol, after having been opsonized with C3 in the extracellular environment.^21^, ^22^ In these cases, entry of C3 into the cytosol is clearly facilitated by the invasive nature of microbes, which are able to escape from within endosomes or phagosomes by penetrating the lipid bilayer surrounding these organelles. However, cell‐intrinsic C3 has also been shown to have a homeostatic role in autophagy regulation, by interaction with ATG16L1,^24^ a cytosolic protein. In this case, re‐addition of C3‐ to C3‐deficient cells was unable to rescue their autophagy‐deficient phenotype, demonstrating a requirement for cell‐intrinsic C3 expression, possibly because this function is mediated by cytosolic C3; cellular uptake of proteins by endocytosis usually limits access of those proteins to the lumen of membrane‐enclosed vesicles, without access to the cytosol and cytosolic proteins such as ATG16L1. This hypothesis then necessitates the presence of endogenously expressed C3 within the cytosol of healthy cells. We have recently addressed two possible routes by which C3 enters the cytosol, and characterized the forms in which it does this.^27^

**FIGURE 1:**
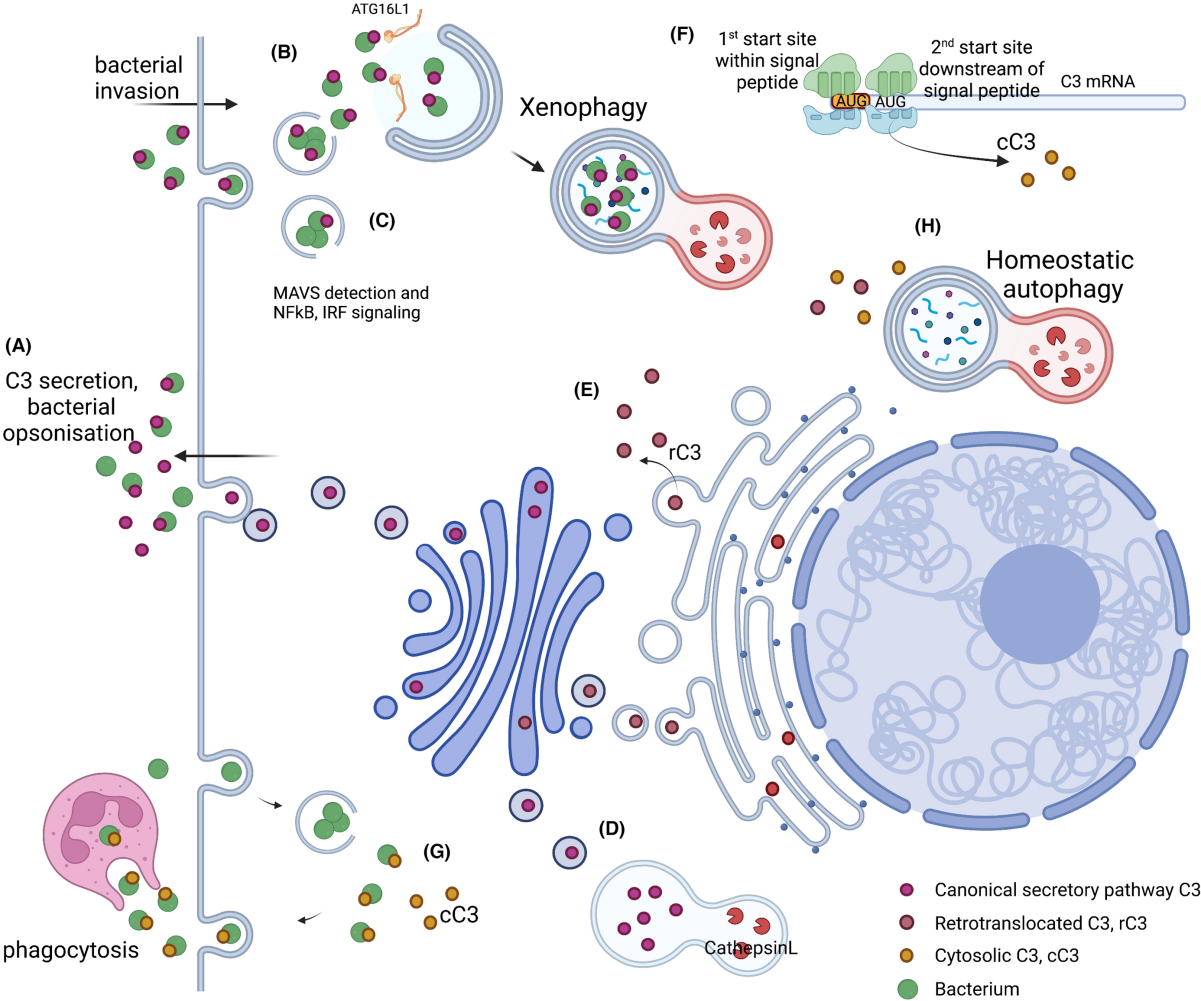
Intra‐ and extracellular sources/routes of C3, proposed and proven. Demonstrated and proposed forms and functions of intracellular C3. Secreted C3 released via the secretory pathway opsonizes extracellular pathogens (green circles, A), which, if not cleared by professional phagocytes, can invade the cytosol of host cells. The deposited C3 is detected intracellularly, by interaction with ATG16L1 (B), leading to autophagy‐mediated bacterial destruction (xenophagy).^22^ Detection of intracellular opsonized C3 also triggers cell‐intrinsic immunity via NFkB and IRF signaling, via MAVS (C).^21^ Cell intrinsic C3 can also be stored within endosomal compartments before cathepsin‐dependent cleavage, leading to C3aR signaling (D), delivering growth and survival signals. Cell‐intrinsic C3 can also enter the cytosol via retrotranslocation from the secretory pathway (E), so‐called retrotranslocated C3 (“rC3”), as well as being expressed from a non‐canonical translational start site downstream of the signal peptide (F),^27^ leading to direct expression into the cytosol, so‐called cytosolic C3 (“cC3”). C3 within the cytosol can also detect and opsonize cytoinvasive *S. aureus* in a non‐canonical fashion dependent on additional cytosolic factors (G),^27^ enhancing subsequent phagocytic killing after the cellular escape. Endogenous C3 within the cytosol can also mediate homeostatic autophagy in pancreatic beta‐cells (H),^24^ although it is not yet addressed whether this is due to rC3 or cC3.

The first investigated mechanism is direct expression within the cytosol. This form of C3 is termed cytosolic C3, or cC3 (Figure 1). The N‐terminal signal peptide of C3 directs the nascent protein into the ER lumen during translation, via interaction with cytosolic factors and the Sec61 pore,^55^ but additional potential in‐frame translation initiation sites also exist, with two additional in‐frame AUG sites present directly after the signal peptide‐encoding region of C3 mRNA.^27^ If protein translation initiation starts here, no signal peptide is translated, and the nascent protein would be expressed directly into the cytosol. The potential of this mechanism was experimentally tested by site‐directed mutagenesis of C3 cDNA,^24^ as well as targeted gene editing,^27^ to mutate the canonical translation initiation codon. In both cases, C3 protein was still expressed, but within the cytosolic fraction of cells, with no C3 found in the secretory pathway or extracellular space. Consistent with the known intracellular sites of C3 post‐translational modification (furin cleavage and glycosylation), this cytosolic C3 was unprocessed, remaining in the pro‐C3 form, and unglycosylated. Interestingly, additional site‐directed mutations of either or both of the in‐frame AUG codons failed to prevent the expression of cytosolic C3,^27^ suggesting that the translational initiation site may be a non‐AUG codon, something now recognized as a common mechanism of regulation of protein expression.^60^ Translation initiation of C3 can therefore occur at a non‐AUG start codon downstream of the signal peptide, delivering the nascent protein directly into the cytosol, where it can interact with cytosolic proteins such as ATG16L1. As described above, the functional relevance of cytosolic C3 was shown in an in vitro model of cyto‐invasive *S. aureus* infection, where the outcome of infection in C3‐KO cells was rescued in ∆AUG1 cells only able to express C3 within the cytosol.^27^ Although the evidence from both C3 cDNA and gene‐edited cells show that C3 can clearly be expressed directly into the cytosol from non‐canonical translation initiation start sites, the exact codon(s) involved, and how their use is regulated, is still under investigation.

### 3.3 C3 retrotranslocation from the secretory pathway into the cytosol

The second investigated pathway is termed retrotranslocation, which describes the retrograde transport of proteins from the lumen of the ER into the cytosol. This pathway is most commonly associated with ER‐associated degradation (ERAD), a vital quality control mechanism by which proteins that fail to fold within the ER lumen are transported through the retrotranslocation channel into the cytosol, where they are ubiquitinylated and degraded by the proteasome.^61^ The removal of proteins from ER to the cytosol can be achieved by the p97/VCP‐NPLOC4‐UFD1L complex,^62^ within which UFD1L recognizes ubiquitinylated substrates. We identified UFD1L as a binding partner for C3 on protein–protein interaction microarrays,^24^ and it is therefore possible that C3 could interact directly and be transported to the cytosol without being ubiquitinylated, and therefore escape subsequent proteasomal degradation. Consistent with this, processed and glycosylated C3 was found in the cytosolic fraction of cells,^27^ suggesting that C3 within the cytosol may have two origins, being both retrotranslocated as well as directly expressed there by use of an alternative translational start site. We termed the retrotranslocated form of C3 as “rC3” (Figure 1).

### 3.4 Other examples of non‐canonical sub‐cellular localization of proteins

There are plenty of examples of proteins that can have non‐canonical intracellular localization. To start with the use of alternative translational start sites, one example that closely mirrors the proposed cytosolic expression of C3 can be found in mitochondrial ribosomal protein L18 (MRPL18).^63^ Mitochondria contain their own ribosomes, although the MRPL18 gene is found within the nuclear, not the mitochondrial genome. The MRPL18 protein therefore contains an N‐terminal signal peptide that directs its trafficking to the mitochondria, rather than to the secretory pathway as found in the C3 signal peptide. Under conditions of cellular stress, global protein synthesis is often halted, while the translation of specific protective factors, such as heat shock factors, continues. Under these stress conditions, MRPL18 mRNA is translated from a non‐canonical CUG codon downstream of the mitochondrial‐targeting signal peptide, resulting in a default cytosolic localization,^63^ exactly as proposed for C3. Within the cytosol, MRPL18 incorporates into the 80S cytosolic ribosome and allows them to engage stress‐response mRNAs. This is just one example of how alternative translation initiation starts sites allow cells to regulate intracellular localization of proteins, produced from the same mRNA, to allow appropriate responses to external stimuli.^64^ Another example is vascular endothelial growth factor A (VEGF‐A), a well‐known secreted regulator of angiogenesis with important roles in development, tissue growth, and cancer. As well as there being VEGF‐A isoforms encoded by different splice variants, an alternative upstream non‐AUG initiation codon also produces C‐terminal extended isoforms, which are processed to produce an intracellular N‐VEGF‐A product^65^ that has a nuclear, rather than secreted localization. VEGF‐A can also signal intracellularly, without being secreted, in a so‐called “intracrine” fashion.^66^ The use of alternative translation initiation codons in VEGF‐A is regulated by changes in mRNA secondary structure induced by cellular stress conditions, and internal ribosome entry sites (IRES),^65^ and a human polymorphism found in the IRES‐B reduces translation initiation from the CUG codon and is linked to various human diseases.^67^ A third example can be found in the well‐known tumor suppressor, PTEN, which is usually found in the cytosol and nucleus, and regulates the cell cycle. Additional N‐terminally extended isoforms can be produced from an in‐frame upstream CUG alternative start codon, which localize to the mitochondria and functions in mitochondrial metabolism,^68^ but has also been shown to be secreted in vivo, where it can be cleaved to produce fragments with extracellular tumor suppressor activity.^69^ These examples demonstrate the complexity of control and variety of possible products that can be produced from one messenger RNA, which is required to allow a diverse repertoire of functions from the surprisingly small number of protein‐coding genes in the human genome.

Apart from splice variants or the use of alternative translational start sites, cell‐type specific mechanisms may exist that lead to individual proteins localizing to different sub‐cellular compartments in different tissues. A recent example of this was described for tissue non‐specific alkaline phosphatase (TNAP), a cold‐inducible protein that plays important roles in adaptive thermogenesis in thermogenic adipocytes. Like CD59, TNAP is usually GPI‐anchored at the cell surface. Surprisingly, when the same cDNA encoding TNAP was introduced into different cell types, TNAP localized to mitochondria specifically in thermogenic adipocytes,^70^ compared to a cell surface localization in other cell types, showing that trafficking of specific proteins produced from the same mRNA molecule can be cell type‐dependent. Therefore, the sub‐cellular localization of a particular protein in one cell type does not necessarily exclude other destinations in other cells.

Other examples of retrotranslocated proteins include CD59. As described above, we have identified splice isoforms of CD59 that reside in the cytosol and mediate insulin secretion.^16^ While these CD59‐IRIS isoforms have novel C‐terminal domains and so are not GPI‐anchored, they have N‐terminal sequences identical to canonical CD59, and therefore also contain signal peptides directing the nascent protein to enter the ER lumen, where they are glycosylated. From here it is expected they would follow the secretory pathway and enter the extracellular space, but we found that they were retained within the cell, and were not detected in cell supernatants. Instead, forms of CD59 lacking a GPI anchor were found within the cytosolic fraction of cells.^15^, ^16^ This retrotranslocation from the secretory pathway to the cytosol was dependent on the N‐terminal glycosylation of CD59.^15^ It is thought that N‐linked glycosylation can be used as a time‐dependent quality control mechanism for protein folding within the ER: initial Glc3Man9GlcNAc2 carbohydrate chains added to N‐glycosylation sites are trimmed down within the ER first by rapid removal of terminal glucose residues, then removal of mannose residues by mannosidase I in a relatively slow reaction,^71^ to allow time for correctly folded proteins to be transported from the ER to further destinations. Misfolded glycosylated proteins that are trapped within the ER instead develop trimmed oligosaccharides that are recognized by ER‐resident ERAD‐associated lectins,^72^, ^73^, ^74^ and are targeted for retrotranslocation to the cytosol.^61^ Consistent with this, removal of the N‐linked glycosylation site prevented CD59 retrotranslocation, and non‐GPI‐linked CD59 found in the cytosol had lower levels of N‐linked glycosylation compared to canonical CD59, indicating trimmed or immature oligosaccharides.^15^ How retrotranslocated CD59 within the cytosol avoids ubiquitinylation and entry into the ERAD pathway, and whether N‐linked glycosylation is also involved in C3 retrotranslocation, is currently unknown.

Another example of a retrotranslocated protein includes calreticulin, a classical ER chaperone protein that is also found in multiple additional cellular compartments.^75^ Calreticulin is retrotranslocated from its canonical location in the ER into the cytosol,^28^, ^76^ in a manner also independent of ubiquitinylation. Cytosolic calreticulin interacts with focal adhesion proteins^77^ and rescues a defective cell adhesion phenotype in fibroblasts of knockout mice,^76^ indicating specific functions of the retrotranslocated cytosolic form. A further example is Nrf1, a transcription factor that regulates the expression of proteasomal subunits, but that is initially expressed within the lumen of the ER; after retrotranslocation via interaction with p97, it is then able to enter the nucleus and act upon target genes.^78^

These examples and many more give precedence for non‐canonical subcellular localizations of secreted proteins, as well as for the existence of alternative and non‐canonical translational initiation sites leading to differing products from the same mRNA molecule. These mechanisms allow specific and sometimes stimuli‐responsive regulation of protein localization, as well as allowing a diversity of function of products from a single gene.

### 3.5 Are intracellular functions of complement compatible with their local environment?

Some aspects of intracellular environments vary widely, including reducing potential and pH, raising questions of whether complement proteins can be expected to behave in a biochemically similar way in the intracellular as compared to the extracellular environment. Endosomes, where pools of intracellular C3 or C3a “stores” have been identified,^30^, ^36^ have an acidic pH of about 6.5 (early endosomes) to 5.5 (late endosomes). Complement convertase activity is slightly increased at pH 6.8,^79^ while activity is similar at pH 6.5 as at pH 7.4.^79^, ^80^ Below this, however, activity quickly becomes inhibited. Therefore, complement convertases can be expected to be active within early endosomes. Cathepsin L is found within the even more acidic lysosomes, being most active at pH 3–6.5, but was reported as being able to cleave C3 into functional C3b and C3a at pH 7.3.^30^ Interestingly, under conditions at pH 6 where no C3 cleavage was found by cathepsin L alone, the addition of complement factor H greatly enhanced C3b production,^32^ showing that uptake of factor H, or endogenous factor H production, could alter the optimal conditions required for intracellular C3 cleavage and C3a production. The fact that the pH of intracellular compartments may not match those in the extracellular environment where we are more familiar with conventional complement convertase formation, should not rule out intracellular functions, which may occur via different mechanisms (e.g., cathepsin cleavage), or by entirely non‐canonical interactions with other proteins (e.g., ATG16L1). The differing pH in different endosomal compartments may also provide an important means of regulation of the activation or cleavage of intracellular complement proteins, as it plays a well‐understood role in regulating the controlled function of other proteins such as the transferrin receptor,^81^ or the neonatal Fc receptor.^82^

The potential presence of complement proteins in the cytosol presents another potential issue, of the reducing nature of this environment. Disulfide bonds are important for the structure and therefore the function of C3 in the extracellular environment, and may be expected to either not be formed during expression in the reducing cytosolic environment, in the case of C3 expressed from an alternative translational start site downstream of the signal peptide, or to be reduced after retrotranslocation from the ER. We incubated serum‐purified C3 with reduced glutathione, the most abundant reducing agent in the intracellular environment, and this was not able to break the inter‐chain disulfide bond within C3 even at concentrations well above those found within the cytosol.^24^ In addition, a substrate‐trapping active site mutant of thioredoxin, another major intracellular redox protein capable of breaking disulfide bonds in target proteins, did not interact with C3,^83^ while it did interact with other complement proteins, C4BP and factor H. The disulfide bonds of C3 may therefore be protected within its folded structure, similar to how the highly reactive thioester group is protected until exposed after cleavage of C3 and conformational change to C3b. Consequently, we found that C3 expressed directly into the cytosol in ∆ATG1 gene‐edited cells was present in a reduced state, while the retrotranslocated form found within the cytosol in wild‐type cells remained oxidized.^27^ While reduction of disulfide bonds prevents canonical functions of C3,^84^ this does not necessarily rule out non‐canonical functions within the cell. Once opsonized onto cell surfaces, C3 does not lose all activity when subsequently exposed to reducing agents,^84^ and the more recently discovered cell‐intrinsic immune responses to cytoinvasive C3‐opsonized pathogens^21^, ^22^ must also be taking place via recognition of opsonized C3 within the reducing cytosol. The structure–function nature of these interactions are not currently understood, but the example of CD59 can again illustrate differing structural requirements for cell‐surface/extracellular and cytosolic/intracellular functions of the same protein, where the human C64Y mutation, which disrupts the formation of a disulfide bond, prevents inhibition of MAC deposition at the cell surface, but does not alter the ability of intracellular cytosolic CD59 to mediate insulin secretion.^15^

### 3.6 A note on protein “Moonlighting”

The hypothesis that the functions of complement proteins, and in particular C3, may be different intracellularly compared to in the extracellular environment, may be met with resistance by those who believe that a particular protein has evolved for one specific defined function. The human genome has a surprisingly limited number of genes for such a complex organism, and the efficiency of this is in fact in part due to exactly this multiplicity of functions of both individual genes as well as proteins. We can again draw attention to the example of CD59, where the intracellular CD59‐IRIS isoforms have entirely different functions, in regulated secretion, to cell surface CD59, in complement inhibition. In mice, the CD59 gene has been duplicated,^85^ allowing further specialization, whereby CD59A has ubiquitous expression and appears to be the major factor in MAC inhibition, whereas CD59B has more limited tissue expression, and encodes the intracellular splice isoforms that mediate insulin secretion.^16^ This reveals the process by which evolution can develop a gene into having multiple functions, followed by duplication and further specialization, rather than the more commonly assumed reverse process (duplication followed by specialization).^86^ Both processes provide an expanded range of molecular activities, although having single genes with multiple functions limits overall genome size, presumably an evolutionarily advantageous trait. Hundreds of other proteins have been identified with diverse multiple functions, many of which are compiled in the searchable online MoonProt database.^87^ Some human examples include calreticulin and VEGF‐A described above, but also include GAPDH, familiar to all high‐school biochemistry students. GAPDH is an enzyme involved in glycolysis but is also an important RNA‐binding protein that regulates inflammatory responses by preventing the translation of specific cytokine mRNAs,^88^ as now shown by numerous publications. The switch to aerobic glycolysis during human effector T‐cell activation is thought to sequester GAPDH in its primary enzymatic role, releasing bound mRNAs and allowing the translation and secretion of pro‐inflammatory cytokines.^89^ GAPDH therefore has two important but competing roles within the same cell. So‐called “moonlighting” is therefore not unheard of among mammalian proteins. Complement and in particular C3 already has many known diverse extracellular roles, leading to C3's description as a molecular “Swiss army knife”,^90^ and a set of intracellular interactions may also be soon added to its known repertoire of functions.

## 4 SOLUTIONS: FUTURE DIRECTIONS AND MODERN METHODS

Historically, the complement field has focused on extracellular complement, where individual complement protein interactions, structures, and functions have been described in exquisite detail.^23^, ^91^, ^92^ However, investigating the world of intracellular complement requires new methods and different skill sets. In addition, the controversial nature of the topic requires a higher standard of evidence to establish the hypothesis, and overturn dogma. In particular, the study of C3 within cellular systems presents challenges, due to the nature of C3 itself, which can exist in several processed forms^90^ and is proposed to be present in various cellular compartments. The introduction of membrane‐permeable complement inhibitors is a first step in being able to determine whether functions of complement can be assigned to an extracellular or intracellular compartment, with the caveat that the interactions and cleavage of intracellular C3 may not necessarily be identical to those found outside the cell, and therefore may not be subject to inhibition by drugs designed to target the canonical complement pathways. Being able to distinguish the different forms of C3 found within cells also requires the use of carefully validated neoepitope‐specific antibodies that can be relied upon to specifically recognize a given cleavage product, for example, C3a, while not recognizing intact, uncleaved C3. Here, we have found that antibodies validated as highly specific for the detection of complement proteins in serum samples do not necessarily give such clean signals when used on cellular samples or cell lysates, and all such experiments should be carefully validated, preferably with CRISPR/Cas9‐KO cells as the “gold standard” of negative controls. Specific antibodies can also be used to determine the origin of individual intracellular C3 species; recently, we raised antibodies against the N‐glycosylation sites of human C3 alpha‐ and beta‐chain, which were purified on peptide‐conjugated columns and then absorbed against immobilized serum‐purified C3. The resulting antibodies only recognize the “naked” glycosylation sites, with the binding being sterically hindered when the site is decorated with attached carbohydrates.^27^ These antibodies therefore recognize and immunoprecipitate cytosolic C3 expressed directly into the cytosol and that is therefore unglycosylated, while they do not interact with canonical C3 from the secretory pathway.

In addition to highly validated antibodies, stricter spatial resolution would also bring higher confidence in localizing intracellular C3, especially when colocalizing with other intracellular proteins. Immunogold labeling coupled with scanning electron microscopy, or super‐resolution microscopy, would be preferable in localizing C3 to distinct organelles, while methods such as proximity‐ligation assays^93^ also narrow co‐localization of proteins down to a proximity of 40 nm, far better than the limit of resolution of conventional confocal microscopy, at about 200 nm.

Since the activity of complement proteins is extensively regulated by proteolysis it is of interest to expand the use of cell‐permeable inhibitors of proteases already known to cleave complement proteins, as well as to screen for novel intracellular proteases targeting complement proteins. This could help in elucidating which processes involve classical interactions and proteolytic steps mediated by complement proteases, and which involve non‐canonical activation. Further, it will be important to determine structural requirements for various functions of intracellular complement proteins such as C3. For instance, is there involvement of the thiolester, or regulation by cleavage of C3a? For example, we observed that ATG16L1 bound with higher affinity to uncleaved forms of C3 (C3, C3met, and C3H_2_O) than to cleaved fragments such as C3b and C3d.^24^ Since it is known that ATG16L1 must again dissociate from autophagosomes before their association with lysosomes, this step may be facilitated by cleavage of C3 that would then decrease its affinity to ATG16L1, allowing its release.^94^

The use of CRISPR/Cas9 for in vitro gene editing has become widespread as a powerful method that is nevertheless accessible, cheap, and requires little expertise beyond traditional cloning and transfection techniques. The basis of this technique is to target Cas9 or other enzymes to specific sites in the genome, where targeted mutations, indels, or even the knocking‐in of tags can be introduced, which are then expressed from the endogenous C3 promoter without the potential artifacts that can be introduced by the overexpression of proteins of interest at unphysiological levels. CRISPR/Cas9 plasmids are widely available at a low price from the distributor Addgene, and protocols and free tools for the design of guideRNAs that target specific genomic sites are widely available online.^95^, ^96^ The application of gene editing can be used to dissect apart the separate potential intracellular species of C3 in several ways. We have used gene editing to target C3 in several cell types so far, introducing single nucleotide indels directly downstream of the canonical translational start site (ATG1) to create the ∆ATG1 cells described above. These cells therefore express only cytosolic C3 and can be compared with WT and KO cells to investigate the dependence of cellular functions on particular intracellular C3 species.

Gene editing can also be used to introduce tags, such as small fluorescent proteins, onto cellular proteins expressed at endogenous levels.^97^, ^98^ This method could be invaluable in investigating and tracking subcellular localization of intracellular C3 species, especially using live imaging to track changes in the localization of intracellular C3 in response to external stimuli, such as fusion of C3‐containing endosomes with cathepsin‐L containing lysosomes that should allow the proposed cleavage to C3b and C3a. As well as creating fluorescent fusions of C3, or for example anaphylatoxin receptors, all expressed at endogenous levels, other functional tags could also be introduced. While gene editing has produced C3‐KO and ∆ATG1 cells, as yet there is no way of producing cells lacking only cytosolic C3, while still expressing canonical C3. The dTAG system is a method for rapidly degrading cytosolic proteins, using small PROTAC (proteasome targeting chimeras) drugs that cross‐link ubiquitinating enzymes with a small protein domain knocked into target proteins.^99^ As these ubiquitinating enzymes are found within the cytosol, treatment with the specific PROTACs should result in inducible degradation of cytosolic protein isoforms, while leaving secreted forms untouched. Similarly, Trim‐Away is a method for rapid degradation of proteins, dependent on Trim21, a high‐affinity cytosolic Fc receptor and ubiquitinylating enzyme.^100^ These methods should therefore also primarily target C3 or other complement proteins found within the cytosol, rather than the secretory pathway. Such methods will be necessary to validate results and assign functions to specific isoforms of C3 from various intracellular or extracellular origins, or subcellular localizations.

Another development that is already allowing better investigation of cell‐intrinsic functions of C3 can be found in the development of the first floxed C3 mouse,^58^ which, when crossed with mice expressing Cre recombinase under a tissue‐ or cell type‐specific promoter, allows the creation of tissue‐specific C3 knockouts. The removal of cell‐intrinsic C3 from specific cell populations that are otherwise exposed to high levels of hepatocyte‐derived C3 in the extracellular environment could answer questions not only about the roles of C3 in specific cell types but also show the relative importance of intrinsic versus extrinsic sources of C3, and therefore by deduction, demonstrate cell‐type specific roles of intracellular C3.

To conclude, recent years have provided much suggested evidence for intracellular functions of complement, but have raised many more questions as to the mechanisms by which canonical and non‐canonical functions of intracellular complement proteins may function. More questions currently remain than answers. More work is required to fully explain and therefore firmly establish the hypothesis of intracellular complement as an important regulator of cellular function and homeostasis. Understanding the contribution of intracellular complement to various physiological and pathological processes will be of great potential future benefit, and could drive the development of new therapeutics that could potentially target mechanisms of intracellular complement function, not only in autoimmunity,^30^ but also in recently identified intracellular complement‐mediated pathological mechanisms in infectious disease,^101^, ^102^ as well as in cancer.^35^, ^42^

## CONFLICT OF INTEREST

The authors declare no conflict of interest.

## Data Availability

not applicable since this is review
